# A fine spatial resolution modeling of urban carbon emissions: a case study of Shanghai, China

**DOI:** 10.1038/s41598-022-13487-5

**Published:** 2022-06-03

**Authors:** Cheng Huang, Qianlai Zhuang, Xing Meng, Peng Zhu, Ji Han, Lingfang Huang

**Affiliations:** 1 School of Forestry, Jiangxi Agricultural University, Nanchang, 330045 China; 2 Shanghai Key Laboratory for Urban Ecological Processes and Eco-Restoration, School of Ecological and Environmental Sciences, East China Normal University, Shanghai, 200041 China; 3 Key Lab of Geographic Information Science (Ministry of Education), School of Geographical Sciences, East China Normal University, Shanghai, 200241 China; 4 Department of Earth, Atmospheric, and Planetary Sciences, Purdue University, West Lafayette, IN 47907 USA; 5 Laboratoire des Sciences du Climat et de l’Environnement (LSCE), CEA CNRS, 91191 Gif-sur-Yvette, France; 6 Institute of Eco-Chongming, 3663 N. Zhongshan Rd., Shanghai, 200062 China; 7 School of Water Resources and Environmental Engineering, East China University of Technology, Nanchang, 330127 China

**Keywords:** Environmental impact, Climate-change mitigation

## Abstract

Quantification of fossil fuel carbon dioxide emissions (CEs) at fine space and time resolution is a critical need in climate change research and carbon cycle. Quantifying changes in spatiotemporal patterns of urban CEs is important to understand carbon cycle and development carbon reduction strategies. The existing spatial data of CEs have low resolution and cannot distinguish the distribution characteristics of CEs of different emission sectors. This study quantified CEs from 15 types of energy sources, including residential, tertiary, and industrial sectors in Shanghai. Additionally, we mapped the CEs for the three sectors using point of interest data and web crawler technology, which is different from traditional methods. At a resolution of 30 m, the improved CEs data has a higher spatial resolution than existing studies. The spatial distribution of CEs based on this study has higher spatial resolution and more details than that based on traditional methods, and can distinguish the spatial distribution characteristics of different sectors. The results indicated that there was a consistent increase in CEs during 2000–2015, with a low rate of increase during 2009–2015. The intensity of CEs increased significantly in the outskirts of the city, mainly due to industrial transfer. Moreover, intensity of CEs reduced in city center. Technological progress has promoted the improvement of energy efficiency, and there has been a decoupling between the economic development and CEs in the city was observed during in 2000–2015.

## Introduction

Greenhouse gas emissions, such as carbon dioxide, contribute to global warming^1^, which is a threat for humans^2,3^. Studies have shown that urban areas account for 2% of the total land area, but produce 80% of the greenhouse gas emissions^4,5^. In 2014, the urban population exceeded 65% of the global population^6^. Cities are the main areas of human activity and greenhouse gas emissions^7^. To cope with global climate change and protect the environment, greenhouse gas emissions have drawn increasing attention^8^. However, urban expansion and population growth have driven increasingly intensified carbon dioxide emissions (CEs) in cities, particularly in China^9^. In recent decades, China has experienced rapid economic growth, environmental damage, and massive energy consumption. In 2014, the CEs of China were 10.29 billion tons, 4.21 times that in 1990, far higher than those of the United States (5.25 billion tons) and other developed countries (https://data.worldbank.org).

To date, extensive validation of CEs and their environmental effects have been conducted^10,11^, and measuring CEs from energy consumption has received increasing attention^12–14^. For example, Lu^15^ quantified and analyzed the impact of CEs from fossil fuel consumption at the national level. Wang^16^ analyzed the driving factors of CEs in China. Numerous studies have been conducted on carbon dioxide accounting. Methods of measuring CEs from various processes are developed. Three methods are widely used for carbon accounting: bottom-up, top-down, and hybrid approaches^17–19^. The top-down method is mainly a production-based accounting approach and is extremely useful for quantifying energy flows between regions and countries^4^. Although the top-down method requires less time and labor, it is not suitable for carbon accounting of small systems. The bottom-up method is used for a consumption-based accounting method, which is based on resource consumption at each step of human activity and life cycle analysis (LCA)^4^. The bottom-up method has a relatively high accuracy, but time-consuming and requires a large amount of data^4^. The hybrid method includes the synthesis of bottom-up, top-down, and other methods, such as environmental input–output analysis (EIOA)/LCA^20^ These integrated approaches are suitable for carbon dioxide accounting when detailed energy data are not available.

Although there is extensive research on carbon dioxide emissions (CEs), they are mainly focused on accounting and analyze the relationship between CEs and socio-economic. However, spatial research on CEs is insufficient. Quantification of fossil fuel CEs at fine spatial resolution is a critical need for climate change research and carbon cycle^21,22^. Moreover, quantifying the changes in spatiotemporal patterns of urban CEs is important to understand carbon cycle and development carbon reduction strategies^23^. Recent years, researchers payed more attention to mapping CEs. With the development of remote sensing technology, carbon monitoring satellites have realized the dynamic monitoring of large-scale greenhouse gas emissions (e.g., greenhouse gas observation satellites, GOSAT). GOSAT's sub-satellite spatial resolution is approximately 10.5 km, and it is suitable for monitoring carbon dioxide concentrations on region and global^24^. So, GOSAT images are not suitable for studying urban-scale CEs. Additionally, nighttime lighting (NTL) data sets are widely used to map CEs^25–28^, as there is a close relationship between NTL and population density, where a higher NTL value indicates greater energy consumption^29,30^. The spatial CEs distribution of existing studies has relied on spatial proxies to downscale the total CEs to a grid. Linear regression and panel regression models are the two most commonly used methods^31–33^. This downscaling method can use multi-source data to obtain the spatial distribution of carbon emissions indirectly^34^. Shi^31^ estimated China CEs distribution base on NTL at province level with a spatial resolution of 1 km. However, the map of CEs lacks the details of sectors. Traditional method of mapping CEs based on NTL have the disadvantages of low spatial resolution, larger than 1 km, and inability to distinguish emission sectors. Moreover, Cai^35^ developed the China high-resolution emission database (CHRED), which was constructed using the bottom-up method and a spatial resolution of 1 km, combined socio-economic data. Most of the existing spatial CEs studies were mapped based on the NTL at a spatial resolution of 1 km. Thus, NTL-based CEs distribution still has limitations. On one hand, the distribution of CEs does not reflect further details of its spatial heterogeneity at the city level. Low spatial resolution cannot satisfy the application of fine urban carbon management and emission reduction strategy development. On the other hand, the existing downscaling method of CEs does not distinguish sectors CEs. Zhang^36^ developed an approach for mapping urban CEs based on land use data, the method could distinguish sectors CEs. Moreover, this method cannot express the heterogeneity of CEs for each land use type. Shi^37^ have explored the relationship between NTL and CO_2_ emissions from different industries, such as service industry CO_2_ emissions (SC), traffic CO_2_ emissions (TC), and secondary industry CO_2_. They point out that Suomi NPP-VIIRS data are better suited to revealing CEs from different sectors. Although the method and Suomi NPP-VIIRS data helps to reveal the CEs of different sectors, its spatial resolution is still 1 km. To improve the spatial resolution of CEs, researchers have set up monitoring sites to accurately monitor CEs, but the human and capital costs are high^38^. Therefore, this method not suitable for large scale study. In recent years, emerging big data has provided an opportunity to study the spatial characteristics of high-resolution urban CEs. Web crawler technology (WCT) can be used to obtain information about the location and attributes of the CEs. In addition, points of interest (POI), which contain multi-attribute information and location, is an important data source. For example, Gao^39^ used POI to mapping urban carbon emissions with a high spatial resolution. In addition, power point and industrial facilities were used to map high resolutions CEs^40^. However, studies on high spatial resolution and spatial distribution of CEs in more sectors are still insufficient. In summary, existing CEs distribution research focus on region and global, based on NTL with 1 km resolution. So, urban and fine resolution CEs data is still rare.

To meet the critical demands of carbon cycle study, urban precision carbon reduction strategies and carbon management, in this study, we developed an improved method and mapped urban CEs with a fine resolution by POI data. The CEs distribution of this study has better accuracy and fine resolution, compare to traditional NTL-based method. Moreover, different sectors CEs distribution is clear. We selected the international city of Shanghai as an example to map the CEs. In this study, we used POI data and GIS technology to map the fine resolution urban CEs. First, according to data availability and accuracy requirements, this study applied the bottom-up method to account urban CEs for residential, tertiary, and industrial sectors from 2000 to 2015. Moreover, this study used the POI data and multi sources data which obtained by web crawler technology (WCT). The method of mapping CEs in this study could clearly identify the CEs spatial patterns of each sector, and different from existing studies. We analyzed the spatial patterns of CE in Shanghai. We believe that this methodology could be applied in other fast-growing cities to understand carbon cycle and develop accuracy urban carbon reduction strategies^41^.

## Study area and materials

### Study area

Shanghai is one of the megacities in the world. At the end of 2018, there were more than 24.24 million people living in Shanghai within the area of 6340.5 km^2^. It lies at the front edge of the Yangtze River Delta (YRD). The gross domestic product (GDP) increased more than 31 times from 1990 to 2015. At the same time, rapid urbanization and industrialization caused a sudden surge in energy consumption and an increase in the urban population. The development of the social economy, expansion of the city, and an increase in the population have led to an increase in CEs. Balancing environmental change and socio-economic development is an urgent need for many cities, particularly in mega cities such as Shanghai. As a resource-limited city, Shanghai, its energy mainly depends on imports. Therefore, understanding the distribution of CEs and reducing energy consumption are important ways to reduce urban CEs and achieve sustainable urban development. The findings and methods from this study will help conduct analyses for other cities, particularly developing cities.

### Materials

To estimate the CEs distribution in Shanghai, geospatial and statistical data were used. Geospatial data included POI and land use data. The POI data for 2000, 2005, 2010, and 2015 were in vector format. This was obtained from the Gaode map (https://ditu.amap.com/) using Application Programming Interface (API) technology. We used 2002 POI data replaced the year of 2000, because Gaode was founded in 2001 and provided services in 2002. The POI data includes the residential, industrial, and tertiary sectors points. Residence community (RC) POI and industrial enterprise (IE) POI contain residential location information and type of enterprises, respectively. The residential area was estimated using statistical data from the Shanghai Statistical Yearbook (SSY) and the website of residential rental companies (e.g., https://sh.lianjia.com/) by WCT. The land use data were in raster format with a spatial resolution of 30 m, they were obtained from the Resource and Environment Data Cloud Platform (REDCP, http://www.resdc.cn/AchievementList.aspx). The land use data included cropland, forest, grass, water, construction land, and unutilized land. Statistical data included the energy balance tables (EBTs) of Shanghai, population (POP), GDP, resident consumption, and energy consumption of key energy-consuming industrial enterprises (ECKEIE, Table 1) of Shanghai. These data were obtained from SSY and are available online (http://www.cnki.net/). The EBT contains data on more than 15 types of energy consumption from industrial, tertiary, and residential sectors.

**Table 1:** Energy consumption and carbon emissions weights of key energy-consuming industrial enterprises in Shanghai.

Industrial type	National average energy consumption per 10,000 yuan (ton of standard coal)	Carbon emissions factor (%)
Oil and gas extraction industry	4.59	7.78
Food processing industry	1.17	1.98
Food manufacturing	0.71	1.20
Beverage manufacturing	0.79	1.34
Tobacco processing industry	0.13	0.22
Textile industry	0.99	1.68
Clothing and other fiber products manufacturing	0.26	0.44
Wood processing and bamboo, rattan, palm and grass products industries	1.6	2.71
Paper and paper products industry	2.05	3.47
Petroleum processing and coking industry	3.92	6.64
Chemical raw materials and chemical products manufacturing	4.2	7.12
Pharmaceutical manufacturing	0.56	0.95
Chemical fiber manufacturing	0.88	1.49
Rubber products industry	0.49	0.83
Plastic products industry	0.7	1.19
Non-metallic mineral products industry	5.58	9.46
Ferrous metal smelting and rolling processing industry	4.64	7.86
Non-ferrous metal smelting and rolling processing industry	3.27	5.54
Metal products industry	0.52	0.88
General machinery manufacturing	0.48	0.81
Special equipment manufacturing	0.58	0.98
Transportation equipment manufacturing	0.18	0.31
Electrical machinery and equipment manufacturing	0.11	0.19
Electronics and communication equipment manufacturing	0.09	0.15
Production and supply of electricity, steam and hot water	9.28	15.73
Gas production and supply industry	5.93	10.05
Tap water production and supply industry	5.3	8.98

The data source is Shanghai Statistics Bureau. The weight calculated according to percentage of national average energy consumption.

## Methods

### Estimating carbon dioxide emissions

The CEs of industrial, tertiary, and residential sectors at the year-end were estimated based on the EBT. We selected 15 types of primary energy sources from the energy balance sheet as the accounting source of carbon dioxide. These included raw coal, clean coal, briquette, other coal washing, coke oven gas, coke, other gases, kerosene, crude oil, diesel, gasoline, liquefied petroleum gas, fuel oil, natural gas, and refinery dry gas. The CEs for each sector were estimated according to the number of energy product consumption, carbon content per unit energy calorific value, oxidation rate during energy combustion, and carbon dioxide emission factor (Eq. 1)^42^.

1 $$E=\sum_{j=1}^{n}{C}_{j}\times {I}_{j}=\sum_{j=1}^{n}{C}_{j}\times {L}_{j}\times {P}_{j}\times {O}_{j}\times (44/12)$$

where *E* is CEs, *C* is energy consumption, *I* is carbon dioxide emission factor, *L* is energy low calorific value, *P* is the carbon content per unit energy calorific value, *O* is the oxidation rate during energy combustion, *j* is the energy type, *n* is 15, which indicates the number of energy types, and *44/12* is the conversion coefficient of carbon into carbon dioxide.

### Mapping carbon dioxide emissions

To understand the spatial change in CEs, we mapped the CEs at the urban scale based on GIS Weighted Point Density (WPD) and downscaling theory. Point density mapping has been widely used in spatial mapping research^43^. The density of any point depends on the statistical characteristics of all points in the search range around the point. In addition, WPD estimates the density based on statistical characteristics and weight of point^44^. In this study, we used the WPD to calculate carbon dioxide emission intensity index (CEI). The CEs of each grid cell was mapped according to the CEI based on downscaling method. The distribution of CEs for each sector was mapped according to Eq. (2).

2 $$E=\frac{D\times C}{\sum_{k=1}^{n}{D}_{k}}$$

where *E* is the CEs, *D* is the CEs density, *C* is the total CE, *k* is the index of the pixel, *n* is the count of pixels with CEs density larger than 0.

The CEI reflect energy consumption and CEs intensity. And the CEI of grid cell for each sector was calculated according to Eq. (3) and WPD method^44^.

3 $$D=\frac{\sum_{j=1}^{m}{W}_{j}}{S}$$

where *D* is the CEs density (CEI), *W* is the weight, *S* is the search radius, *j* is the index, and *m* is the total number of pixels in the search area.

In order to map CEI, different weights were used for residential, industrial, and tertiary points. Weights of residential and industrial were calculated according to Eq. (4), and based on residential area and ECKEIE, respectively. Due to the lack of data on tertiary energy consumption intensity, we assume that the weights of tertiary points are the same. Here, we used “1” as the weight. Residential CEs are closely related to household population structure. Since it is difficult to obtain household population structure data in each house. So, residential area was used as the weight of residential CEs allocation in this study.

4 $${W}_{j}=\frac{{A}_{j}}{\sum_{i=1}^{n}{A}_{i}}$$

where *W* is the weight, *A* is the residential area or ECKEIE, i *and j* are the index of POI, and *n* is the count of POI of a sector.

**Figure 1:**
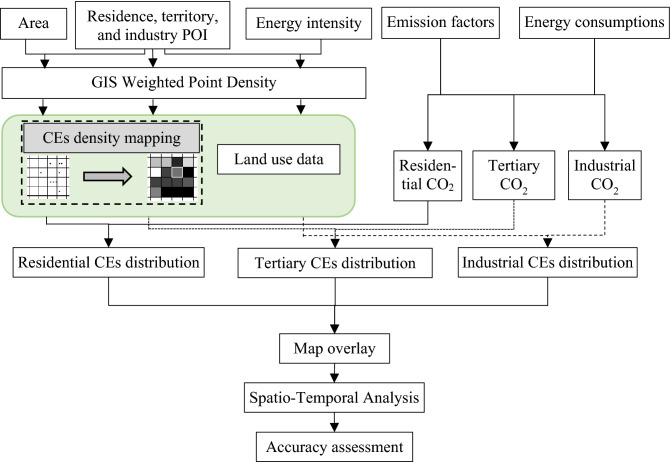
Flow-process of mapping carbon dioxide.

According to the above-mentioned equations used to calculate the energy consumption density for the residential, tertiary, and industrial sectors, mapping the CEs (see Fig. 1) involved combining the CEs calculated from Eq. (1). As shown in Fig. 1, the urban boundary was extracted from land use data, and the boundary was used to clip the energy consumption intensity map, and the CEs boundary was the same as the urban boundary. We allocated CO_2_ according to the energy consumption intensity for each sector, mapping the CEs, and then calculated the total carbon dioxide by overlay analysis.

Due to the lack of available high-resolution carbon emission spatial data as precision validation data, this study adopted the widely used NTL based downscaling carbon emission allocation method to produce validation data. More detail about the method was published by^42^.

## Results

### Changing patterns of CEs

**Figure 2:**
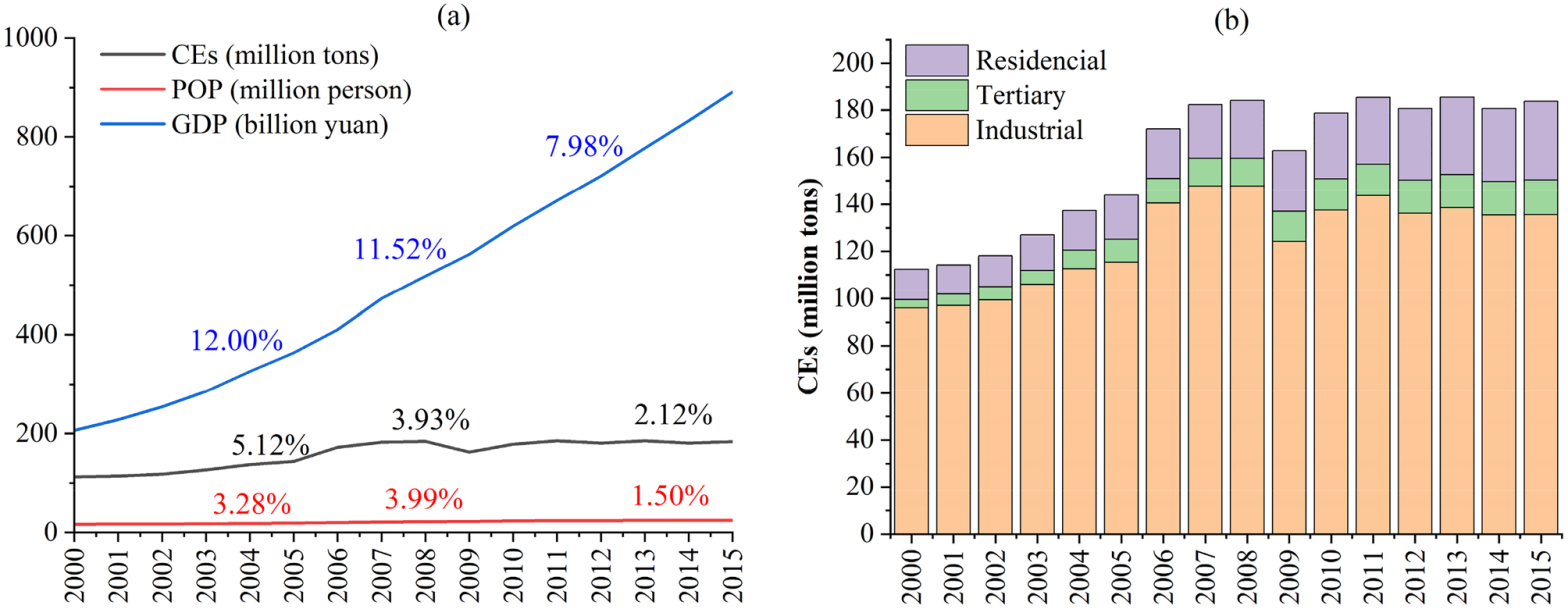
Changes in CEs of Shanghai from 2000 to 2015: (**a**) The growth rate of CEs, population (POP), and GDP. The percentage is the average annual growth rate; (**b**) CEs from industrial, tertiary, and residential energy sectors.

The total CEs in Shanghai increased by 64% from 112.36 to 183.75 Mt, 2000–2015, with a 50% and 331% increase in the population and GDP, respectively (Fig. 2a). The GDP growth rate gradually decreased from 12 to 7.98% during 2000–2015. Similarly, the rate of increase of total CEs decreased from 5.12 to 2.12%. The population growth rate increased from 3.28 to 3.99% from 2000 to 2009 and then dropped to 1.5% from 2009 to 2015 (Fig. 2a). Typically, the GDP growth rate increases faster than the CEs growth rate. A relative decoupling of CEs and GDP can be observed, and this decoupling gradually strengthens (Fig. 2a). In this study, the total CEs comprised of three parts: industrial, tertiary, and residential consumption (household consumption) (Fig. 2b). Industrial CEs increased from 95.9 million tons to 135.58 million tons, but its percentage decreased from 85.35 to 73.79% during 2000–2015. The residential CEs increased from 11.46 to 18.14%. The total CEs dropped significantly in 2009 owing to a decrease in industrial CEs. In 2009, industrial CEs decreased by more than 15%, likely because of the global financial crisis in 2008^45^. The global financial crisis triggered a global recession, which had an impact on the economy in Shanghai as well. The total CEs decreased by 11.56%, and the average growth rate of industrial CEs was 1.60% during 2009–2015, mainly due to the global financial crisis.

### Changes in the spatial pattern of CEs

**Figure 3:**
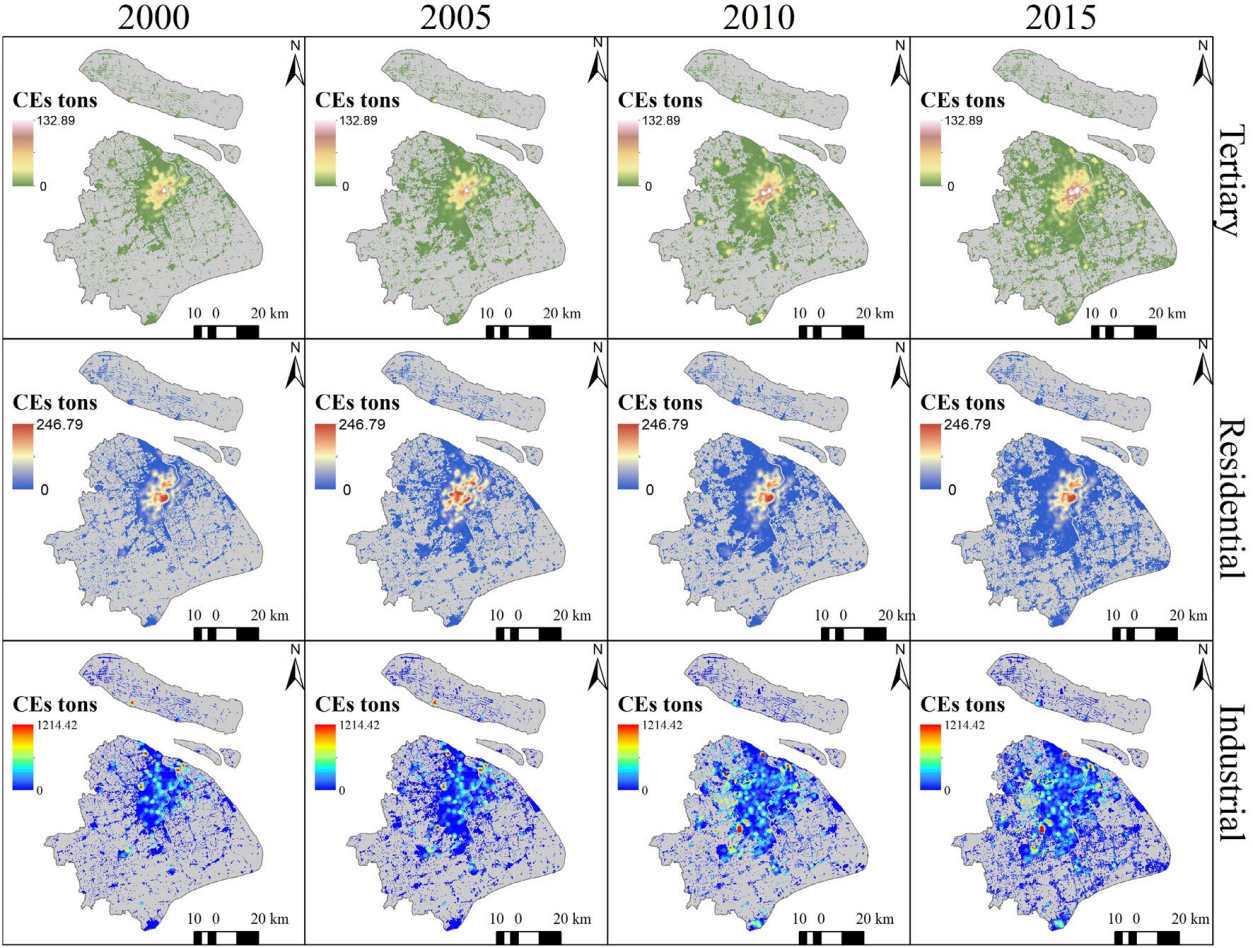
CEs of sectors (created by QGIS version g 3.24.2 https://www.qgis.org/en/site/, Shanghai boundary map obtained from Resource and Environment Science and Data Center https://www.resdc.cn/Default.aspx).

**Figure 4:**
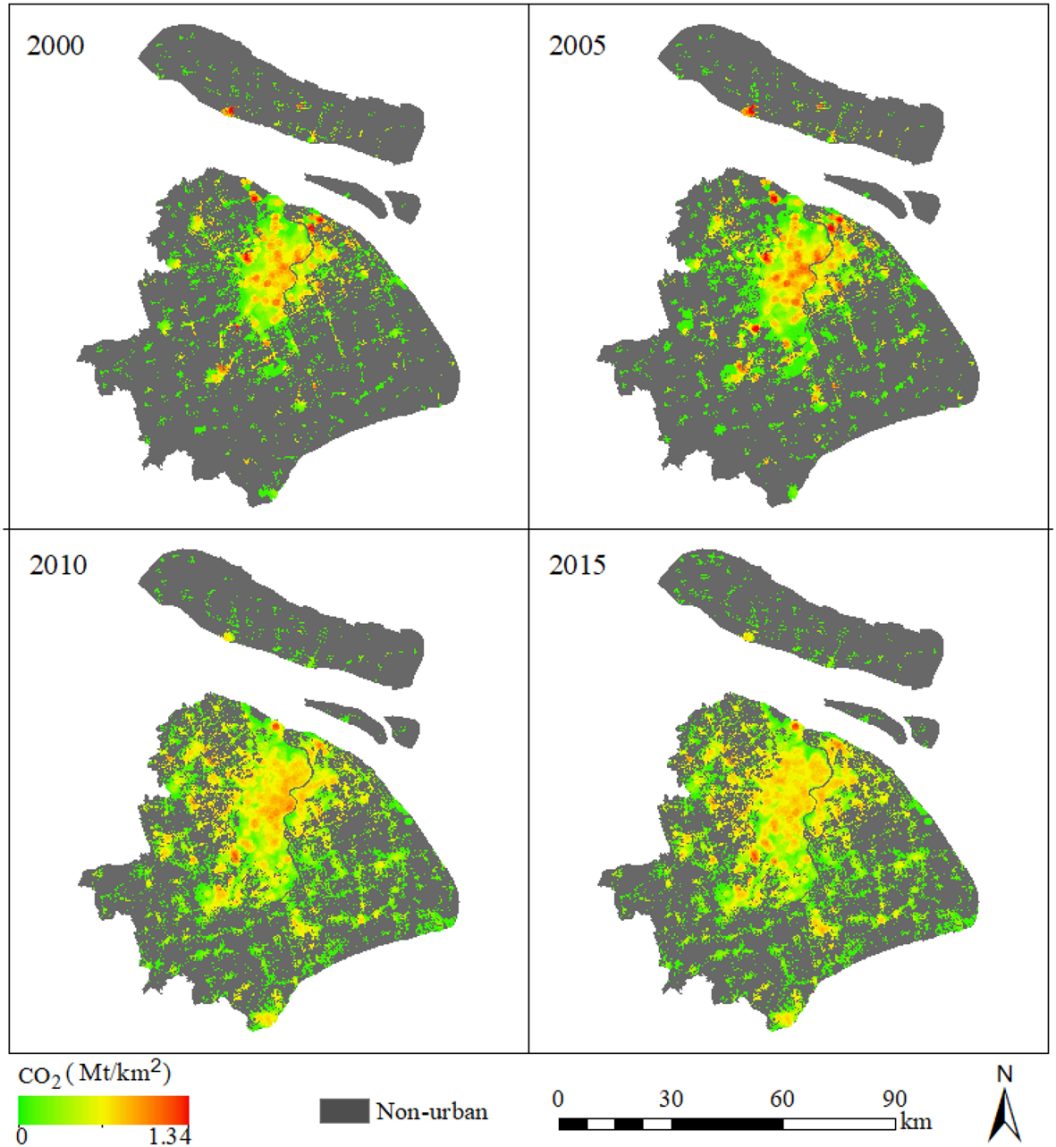
Distribution of CEs as the sum of emissions from the industrial, tertiary, and residential sectors (created by QGIS version g 3.24.2 https://www.qgis.org/en/site/, Shanghai boundary map obtained from Resource and Environment Science and Data Center https://www.resdc.cn/Default.aspx).

Figure 3 shows CEs from tertiary, residential, industrial sectors, from 2000 to 2015. And Fig. 4 shows the total CEs of Shanghai from 2000–2015. CEI increased in the city center area from 2000 to 2005, with the highest CEI increasing from 0.62 to 0.66 Mt/km^2^ (Fig. 4). However, a turning point appeared in 2005, when the CEI was reduced with the expansion of urban areas. In 2015, CEI continued to decrease. In spite of the decreasing CEI in the city center, we take the area within 10 km of Shanghai People's Square as the center of the city. The total CEs increased by 39.75 million tons from 2005 to 2015, and city expand 476 km^2^. In contrast, the CEI of the non-city center area increased significantly from 2005 to 2015. Thus, we concluded that urban expansion and industrial relocation together reduced the CEI in the urban center, mainly due to the shift of industrial enterprises from urban center to urban suburbs. However, total CEs continued to increase.

**Figure 5:**
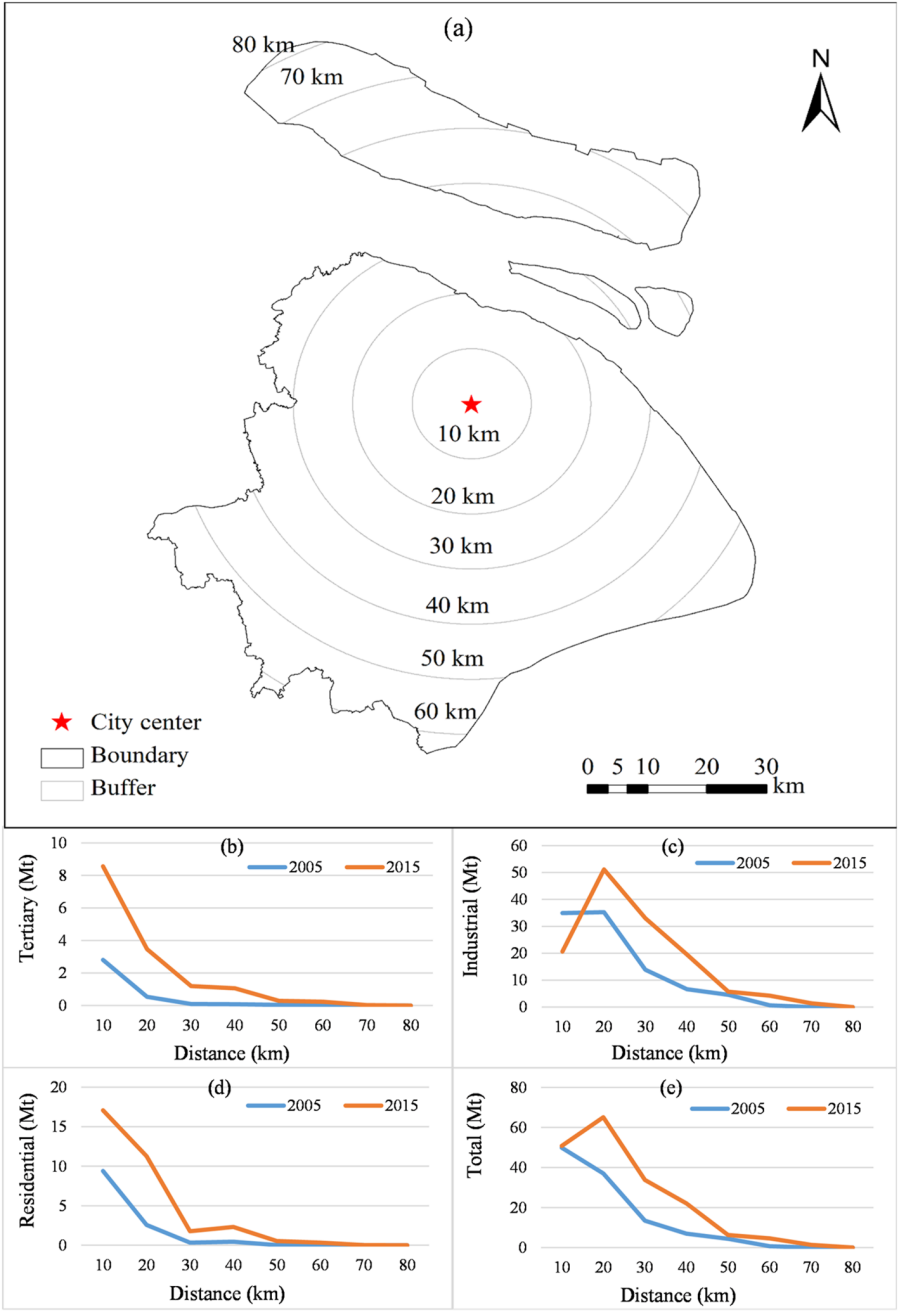
Buffer and CEs summary: (**a**) diagram of buffer; (**b**) the tertiary CEs within different buffer zones; (**c**) the industrial CEs within different buffer zones; (**d**) the CEs of residential sectors within different buffer zones; and (**e**) the total CEs within different buffer zones (created by QGIS version g 3.24.2 https://www.qgis.org/en/site/, Shanghai boundary map obtained from Resource and Environment Science and Data Center https://www.resdc.cn/Default.aspx).

To analyze the change in spatial patterns of CEs between 2000 and 2015, we applied the city center as the center of circle, then we drew multi buffer zones for each 10 km and counted the CEs within each buffer zone (Fig. 5a). From 2000 to 2015, the total CEs increased to 50.79 Mt (1.76%) in the city center (within 10 km). However, a remarkable increase (75.81%) was observed in peri-urban areas (distance to the city center between 10 and 20 km, Fig. 5e). Moreover, CEs increased rapidly in the suburbs (the regions which distance to the city center from 20 to 70 km). This indicates that human activities spread towards peri-urban areas with urban expansion. When examining industrial CEs, we found that CEs significantly reduced (41.05%) in the city center areas. We also observed an increase (19.12 Mt, 138.09%) in the suburbs (distance to the city center from 20 to 30 km) from 2000 to 2015 (Fig. 5c). Additionally, industrial CEs in the areas between 30 and 70 km from the urban center generally increased rapidly (Fig. 5c). This could be explained by industrial transfer, where in the industries were moved to peri-urban areas of Shanghai and other provinces^46^. During the “Twelfth Five-Year Plan” period, due to the continuous increase in land and labor costs, tertiary costs, the pressure of resources, environment, and the transformation of its economy and industrial structure, industries shifted to the suburbs of Shanghai and nearby cities to achieve a cross-regional industrial division of labor. This trend is becoming increasingly apparent (http://www.shanghai.gov.cn). In addition, industrial transfer was mainly associated with high energy consuming factories, whereas labor-intensive factories remained in the city center of Shanghai, such as iron and steel enterprises. Thus, while the CEs increased in non-city center areas, they decreased in the city center areas (http://www.shanghai.gov.cn). Interestingly, the residential CEs increased in both the city center and its surrounding areas, particularly in the western part of the city center (Fig. 5d). The largest increase (8.70 Mt) in residential CEs was observed in peri-urban areas (distance to the city center from 10 to 20 km), followed by the city center areas (7.67 Mt). There was a rapid increase in the suburbs situated at a distance of 30–70 km from the center. This clearly indicates that CEs increased with urban expansion between 2000 and 2015. In addition, compared to 2000, population density and human activity intensity also increased in the city center. The CEs from the tertiary sector mainly increased in the city center (5.75 Mt) at a rate of 204.58% (Fig. 5b), primarily as the tertiary sector located in the city center and the intensity of tertiary activity has increased. In addition, the suburbs (distance to the center from 50 to 60 km) showed the fastest growth, followed by the areas located 30 to 40 km from the center.

The intensity of CEs is increasing from urban centers to suburbs, and the spatial distribution area of CEs has increased significantly. This is due to industrial transfer and economic transformation and upgrading. The analysis of the urban spatial morphology (Table 2) shows that from 2000 to 2015, the spatial distribution of the city increased in connectivity, agglomeration, they indicate that Shanghai is more compact. The spatial characteristics of CEs are consistent with the urban form. Compact cities are conducive to the development of public transportation and change people's travel behavior, thereby reducing carbon emissions^47,48^. Moreover, reasonable land use and functional allocation can effectively reduce carbon emissions.

**Table 2:** Shanghai spatial form index (SFI).

SFI	2000	2005	2010	2015
CONTAG	46.87	46.88	56.74	57.84
SPLIT	4.53	4.53	2.18	2.20

CONTAG is contagion index, high value is high connectivity; SPLIT is splitting index, high value is more broken and looser. CONTAG and SPLIT were calculated by Fragstas 4.2.1.

### Accuracy evaluation

**Figure 6:**
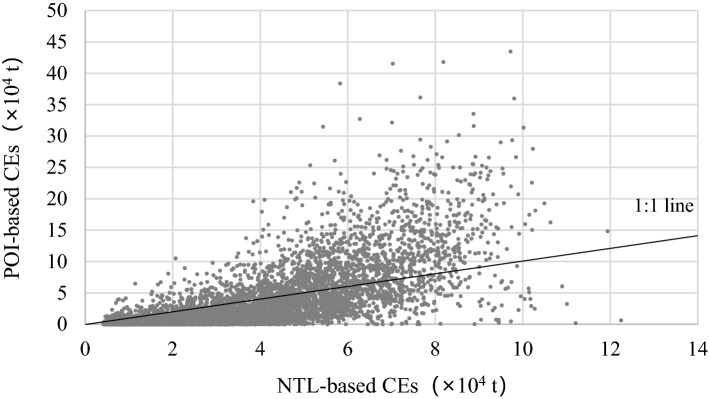
The scatterplot of carbon dioxide emissions by the traditional method (based on NTL data) and this study (based on POI data).

In order to evaluate the accuracy of CEs estimation under this study (POI-based CEs), we used CEs obtained by the NTL-based model method (NTL-based CEs), which maps CEs based on statistical model and NTL data, and then compared to POI-based CEs (Fig. 6). There is a lack of fine-resolution spatial data on CEs, even though satellite platforms such as GOSAT directly observe CEs. The NTL-based model is a good method for mapping CEs at the regional scale, with a resolution of 1 km. The NTL-based CE was produced according to Meng^42^ and employed as a standard for assessing CEs in this study. The resolutions of POI-based CEs and NTL-based CEs are 30 m and 1 km, respectively. We employed 1 × 1 km grids to extract CEs from the two methods, and then mapped them using a scatterplot, as shown in Fig. 6. Also, it is a external validation base on traditional NTL downscaling method.

The results shown that more than 31.6% of the pixel values based on this study method are higher than those based on the traditional NTL method. The highest value in NTL-based CEs was 122.49 kt. We set the low range as 0–30% of the highest value, which from 0 to 36.75 kt. Moreover, the high range is 80–100% of the highest value, which between 85.75 and 122.49 kt. According to Fig. 6, there are more than 40.89% of the total pixels in the low range, the values of NTL-based CEs were larger than POI-based. Additionally, in the high range, more than 15.20% of the total pixels’ value of POI-based CEs larger than NTL-based. These results indicate that the NTL-based method underestimated CEs in the high range and overestimated CEs in the low range, compare to the POI-based method, which may be caused by the spillover and saturation effects of NTL data.

**Figure 7:**
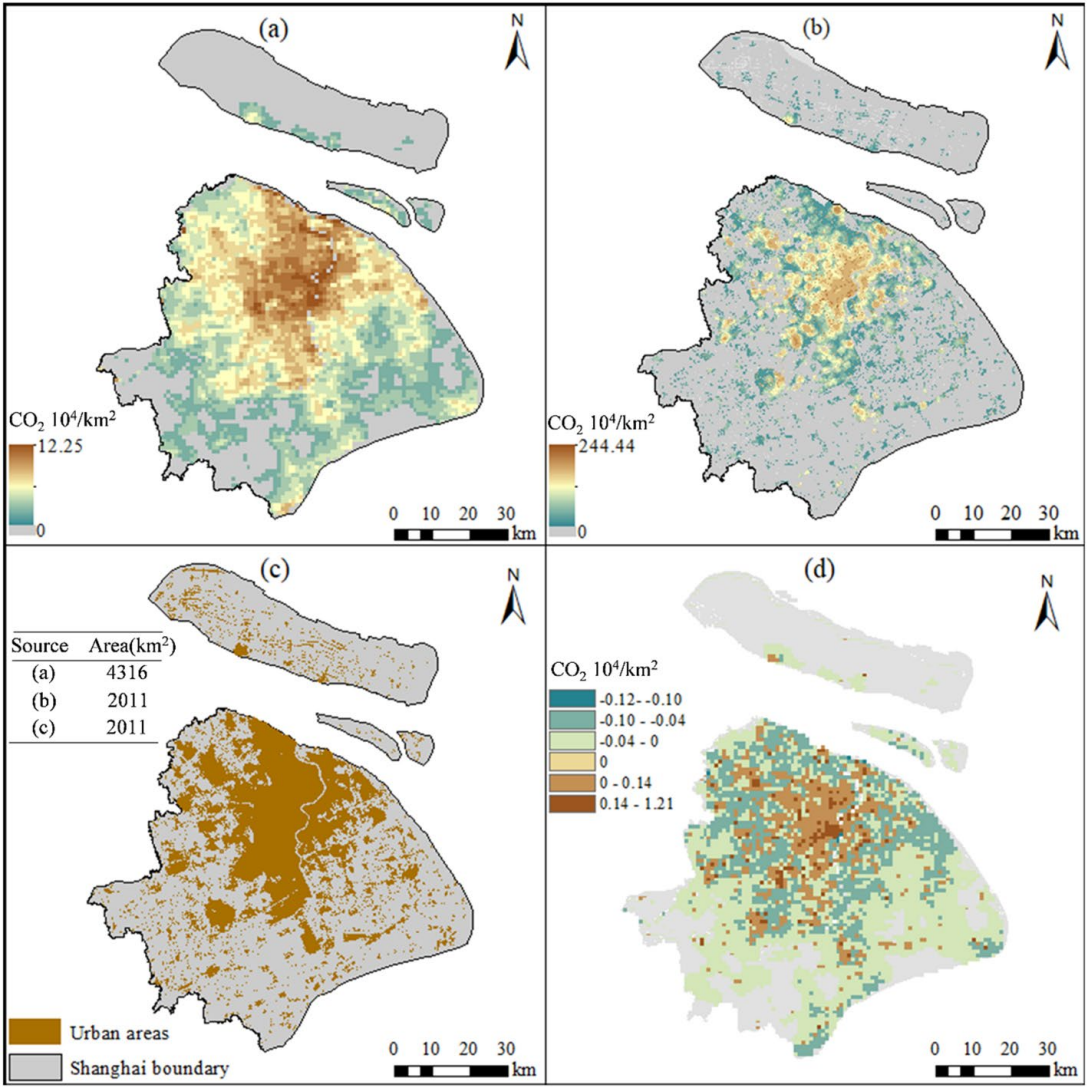
Comparison between the distributions of POI-based and NTL-based methods for the year 2010. (**a**) NTL-based CEs distribution, with a spatial resolution of 1 km, (**b**) POI-based CEs distribution, with a resolution of 30 m, (**c**) urban area counted from land use data, and compare urban area with distribution area of POI-based and NTL-based CEs, (**d**) the differences in value between POI-based and NTL-based CEs (**b–a**); note that we resampled (**b**) to 1 km and then calculated the value (created by QGIS version g 3.24.2 https://www.qgis.org/en/site/, Shanghai boundary map obtained from Resource and Environment Science and Data Center https://www.resdc.cn/Default.aspx).

Figure 7 compares the spatial distribution of CEs based on this study method and traditional method (based on NTL) in 2010 (Fig. 7a–c). There is clearly to known that distribution area of CEs based on the traditional method is significantly larger than that based on this study method. (Fig. 7a,b). The reason is the overflow effect and resolution of NTL data. Moreover, the distribution area of CEs base on this study (POI-based) is close to urban area which extracted from land use data. Because of the CEs mapping method in this study uses urban extent from land use data to correct the distribution extent of CEs. CEs base on this study are higher than those based on traditional method in the inner city, while the opposite is true in the suburbs. (Fig. 7d). This indicating that the traditional NTL-based method underestimates CEs in high emissions areas and overestimates CEs in low emission areas, compare to POI-based method.

**Figure 8:**
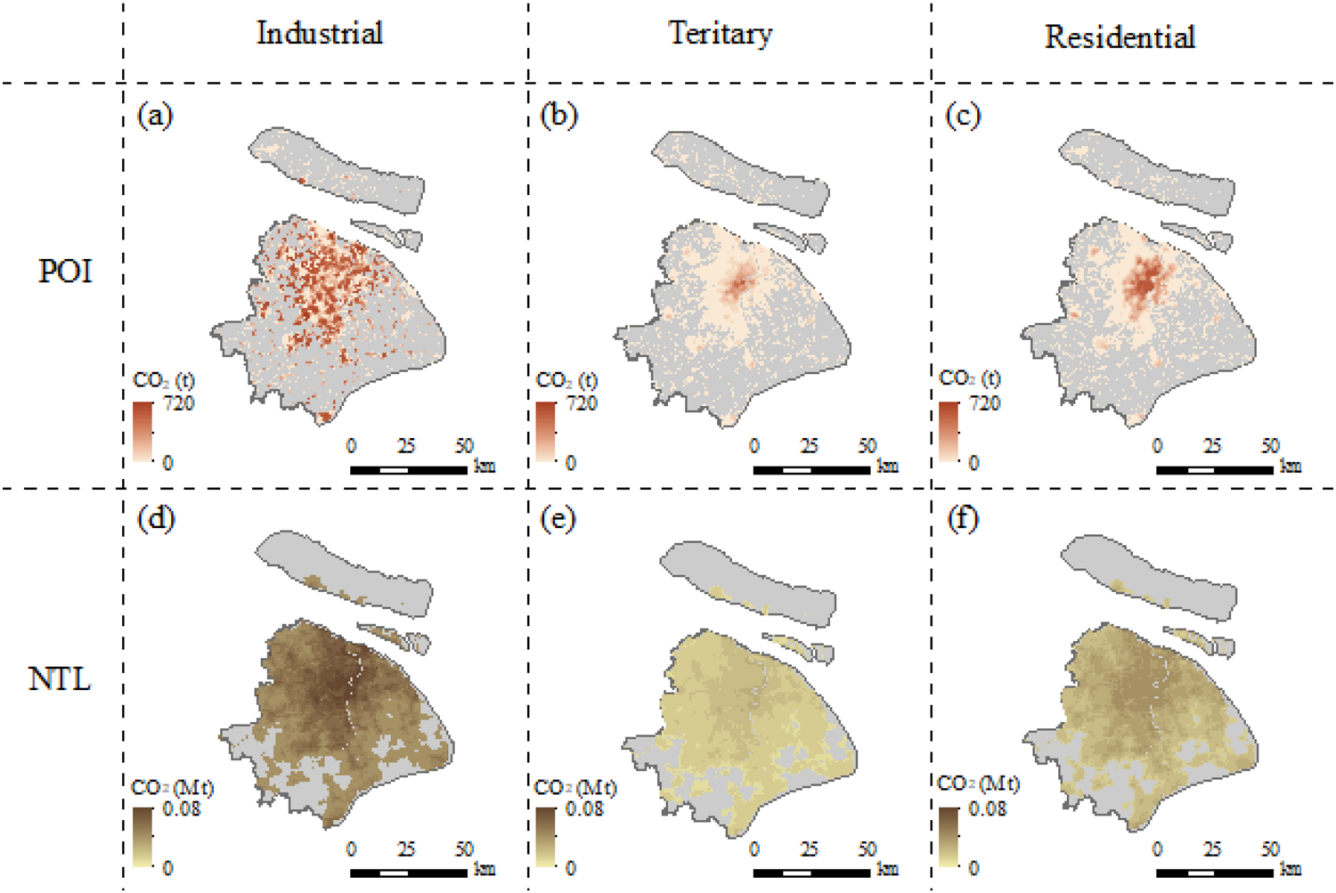
CEs distributions in the industrial, tertiary, and residential sectors under this study (POI) and the traditional method (NTL). (**a–c**) are the spatial CEs estimated by POI method, and (**d–f**) are the spatial CEs estimated by NTL method, and for industrial, teritary, and residential sectors respectively (created by QGIS version g 3.24.2 https://www.qgis.org/en/site/, Shanghai boundary map obtained from Resource and Environment Science and Data Center https://www.resdc.cn/Default.aspx).

We compared the CEs distribution of NTL-based and POI-based method, the CEs distribution of POI-based was found to have more details and fine resolution. Additionally, the heterogeneity of CEs distribution in the three sectors could be assessed at a fine resolution (Fig. 8). The spatial distribution range and characteristics of industrial (Fig. 8d), tertiary (Fig. 8e), and residential (Fig. 8f) CEs based on traditional method are similar and consistent with the NTL data. In contrast, the spatial patterns of CEs by POI-based is different from each sector, and the spatial heterogeneity of CEs for each sector could be accurately assessed (Fig. 8a–c). This is impossible with traditional methods.

**Figure 9:**
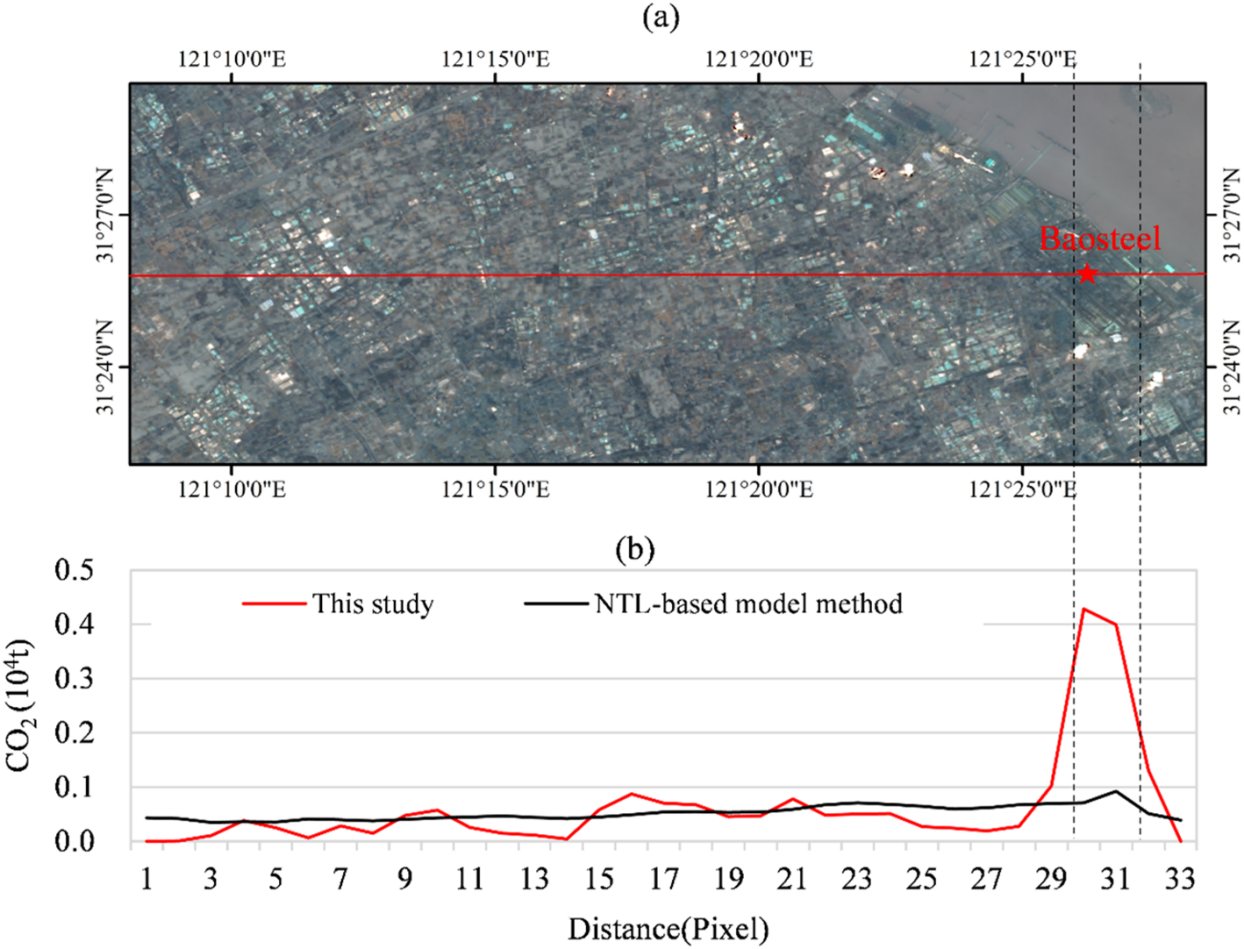
Comparison of CEs obtained from the POI-based (this study) and NTL-based methods. (**a**) Schematic diagram of sampling line; (**b**) comparison of the pixel values at the sampling line using the two methods.

To verify accuracy of CEs by POI-based method, we employed a sampling line (see Fig. 9a) to extract CEs for the two methods, and compared the values (see Fig. 9b). It is worth noting that Baosteel, a steel factory in Shanghai, which is a high emission source, is located at the sampling line. The POI-based CEs (this study) correctly showed a sudden change and reflected the associated emissions, but the NTL-based method not significant (Fig. 9b). In addition, the CEs of NTL-based is a smooth data line compare to CEs of POI-based. The latter has significant difference, this basically consistent with facts. Because of the sampling line passes through the built-up areas and farmland, so the CEs are striking differences.

## Discussion

### Uncertainties and limitations of this method

The distribution of urban CEs was assessed based on POI data and its energy consumption intensity. The uncertainty of the POI data and attribute data was consistent with the uncertainty of the distribution of CEs. In addition, energy consumption data and CEs parameters obtained from the existing literature and materials also have some limitations. As the energy consumed in city areas is imported from other cities and areas, energy use types and energy tastes are different. Therefore, there are limitations in using statistical data and uniform CEs parameters to calculate CEs. Moreover, because of the lack of high-resolution remote sensing data of CEs as reference data, it is difficult to accurately assess the accuracy of our estimations. The lack of more detail on companies', residential, and tertiary energy consumption limits the accuracy of the spatial allocation of carbon emissions. In conclusion, with the improvement of more details of energy consumption data and the enrichment of spatial data, the accuracy and reliability of spatial distribution data of CEs will be significantly improved.

### Potential uses

In this study, a downscaling CEs allocation method based on POI method was developed to achieve fine spatial resolution allocation of CEs and draw spatial distribution maps for different sectors. Based on the fine resolution mapping method of carbon dioxide emissions and dataset at the urban scale, which was proposed in this study, several potential uses could be obtained. They include, but are not limited, the following.

First, this method could be applied to map the distribution of urban CEs and as a decision support tool for the analysis of spatial–temporal changes in high-resolution urban CEs in other cities. Second, high-resolution urban CEs data could be used to understand the relationship between spatial–temporal changes and social economy. Third, fine-resolution urban CEs data could be used as a data source for urban spatial planning, also useful to develop accurate urban carbon management strategies. In addition, fine resolution CEs data could meet the critical need of carbon cycle, climate change research, and emerging flux inversion.

## Conclusions

The goal of this study was to propose an improved method for mapping a fine resolution urban CEs. We found that results obtained using our method had a higher resolution than the traditional NTL-based method. In addition, the method of this study is suitable for mapping the CEs of sectors. This case study in Shanghai suggests that the CEs method based on POI is suitable for the high-resolution spatial distribution under province level. The method effectively draws the spatial characteristics and heterogeneity of urban CEs, and its spatial resolution is better than traditional method. It is important to point out that the POI-based method can accurately determine both emission extent and intensity, be able to identify the carbon emissions of different sectors. In addition, POI-based CEs draws more details and close to reality. Therefore, the improved method is better than the traditional method for mapping CEs. We also found that the total CEs in Shanghai witnessed a three-stage increase from 2000 to 2005, and a steady growth from 2005 to 2009. The global financial crisis strongly impacted CEs growth from 2008 to 2009. The global financial crisis markedly decreased CEs from 2008 to 2009, particularly from the industrial sector. A significant relationship between economic growth and CEs was also observed. The industrial sector was found to be the largest CEs contributor, followed by residential and tertiary sectors. Industrial CEs have shaped the trend in total CEs. These industrial CEs slightly decreased in the city center, but increased in the peri-urban areas. The CEs of residence and tertiary areas significantly accumulated in city center regions and increased rapidly in suburbs.
